# A Comprehensive Review of the Role of Rho-Kinase Inhibitors in Corneal Diseases

**DOI:** 10.3390/life15081283

**Published:** 2025-08-13

**Authors:** Elizabeth Y. X. Leong, Jianbin Ding, Duoduo Wu, Blanche X. H. Lim, Andrea Ang, Evan Wong, Nigel Morlet, Jodhbir S. Mehta, Chris H. L. Lim

**Affiliations:** 1 Department of Ophthalmology, National University Health System, Singapore 119228, Singapore; elizabethleongyx@gmail.com (E.Y.X.L.); ding.jianbin@mohh.com.sg (J.D.); wuduoduo.med@gmail.com (D.W.); blanchelim@gmail.com (B.X.H.L.); 2 Yong Loo Lin School of Medicine, National University of Singapore, Singapore 119077, Singapore; 3 Cornea and Oculoplastics Units, Department of Ophthalmology, Royal Perth Hospital, Perth, WA 6000, Australia; angandrea@hotmail.com (A.A.); evan.n.wong@gmail.com (E.W.); n.morlet@idrs.com.au (N.M.); 4 Singapore Eye Research Institute, Singapore 169856, Singapore; jodmehta@gmail.com; 5 Ophthalmology and Visual Sciences Academic Clinical Programme, Duke-NUS Medical School, Singapore 169857, Singapore; 6 Corneal and External Eye Disease, Singapore National Eye Centre, Singapore 168751, Singapore; 7 Centre for Sustainable Medicine, Yong Loo Lin School of Medicine, National University of Singapore, Singapore 119077, Singapore

**Keywords:** Rho-associated protein kinase, Rho-associated protein kinase inhibitors, corneal endothelium, Fuchs endothelial corneal dystrophy, corneal neovascularization, ocular pharmacology, regenerative ophthalmology, ripasudil, netarsudil, wound healing

## Abstract

There is growing interest in the application of Rho-associated protein kinase (ROCK) inhibitors (ROCKI) to the treatment of corneal diseases. ROCK is a key regulator of several cellular processes in the cornea, including cytoskeletal organization, cell proliferation, migration, inflammation, and wound healing. ROCKI, such as ripasudil and netarsudil, enhances endothelial cell migration, and promotes repair in conditions characterized by endothelial dysfunction. These agents also exert anti-inflammatory, anti-angiogenic, and anti-fibrotic effects for wound healing. As such, ROCKI demonstrate promise as therapeutic options for conditions such as Fuchs’ endothelial corneal dystrophy, pseudophakic bullous keratopathy, and iridocorneal endothelial syndrome. Emerging data further supports ROCKI’s potential in managing corneal neovascularization and supporting recovery following cataract surgery and keratoplasty, reducing the need for donor tissue. This narrative review provides a comprehensive evaluation of ROCKI’s mechanism of action, pharmacological properties, safety profile, applications in corneal disease management, emerging clinical trials, and novel approaches. We emphasize both preclinical and clinical findings, highlight existing evidence gaps, and outline future research priorities.

## 1. Introduction

Rho-associated coiled-coil containing protein kinase (also known as ROCK) is a serine/threonine kinase downstream of Rho guanosine triphosphate hydrolases (GTPases) that regulates actin cytoskeleton dynamics, cell contraction, proliferation, apoptosis, and motility [1]. In the eye, ROCK signalling is widely active and has been detected in corneal epithelium, stroma, and endothelium [2,3,4]. ROCK plays an important role in corneal epithelial homeostasis and wound healing [2]. Pharmacological ROCK inhibition has been shown to accelerate wound closure by enhancing cell migration, proliferation, cell–cell adhesion and cell–matrix adhesion in the corneal epithelium [2]. Within the cornea stroma, ROCK regulates keratocyte contractility and differentiation into myofibroblasts during fibrosis [4]. In endothelial cells, ROCK maintains barrier integrity under physiological conditions, but its excessive activation, such as in response to inflammatory cytokines, can trigger junctional disruption, cytoskeletal contraction, and apoptosis [3].

ROCK signalling functions as a central regulator of cytoskeletal dynamics and cell fate in the cornea, making it an attractive target in corneal disease. ROCK inhibitors (ROCKI), including ripasudil, netarsudil, and Y-27632, target this pathway. Laboratory and early clinical studies suggest that ROCKI can promote corneal endothelial cell (CEC) healing and regeneration, especially in conditions such as Fuchs endothelial corneal dystrophy (FECD) and pseudophakic bullous keratopathy (PBK) [5]. In animal models and select human cases, topical ROCKI, alone or in combination with cell therapy, have been associated with resolution of corneal oedema and restoration of endothelial function [6,7,8].

Given these promising findings, this narrative review outlines the mechanisms, pharmacology, and safety profile of ROCKI, and provides a comprehensive overview of their therapeutic applications across corneal diseases and perioperative care, including post-keratoplasty and post-cataract surgery settings.

## 2. Literature Search Strategy and Review Methodology

A comprehensive literature search was conducted in PubMed, EMBASE and Clinical Trials Gov from inception to May 2025 to identify studies investigating the role of ROCKI in corneal diseases. This search strategy was developed in accordance with the framework laid out by the Scale for the Assessment of Narrative Review Articles (SANRA). Keywords used in the search included but were not limited to: “corneal oedema”, “Fuchs’ endothelial corneal dystrophy”, “pseudophakic bullous keratopathy”, “ripasudil”, “netarsudil”, “ROCK inhibitor”, and “corneal neovascularization”.

In addition to database searching, hand-searching of references from key publications was performed to identify additional relevant studies. Titles and abstracts were initially screened for relevance to the topic. Studies were included if they addressed the pharmacological actions, clinical applications, or safety profile of ROCK inhibitors in corneal or ocular surface disease.

Inclusion criteria encompassed peer-reviewed articles reporting on preclinical or clinical studies in either human or animal models related to the use of ROCK inhibitors in corneal pathology. Articles were excluded if they were not published in English, lacked relevance to ophthalmology, focused on non-ocular applications of ROCK inhibitors, or were duplicate or incomplete records.

Two independent reviewers (J.D. and D.W.) screened all identified abstracts and assessed full texts for eligibility. Differences were adjudicated by the senior author (C.H.L.L.). Key findings were organized thematically and summarized in narrative form and structured tables.

## 3. Mechanism of Action

ROCK is activated when RhoA, in its GTP-bound form (RhoA–GTP), binds to the Rho-binding domain (RBD) of ROCK [9]. This interaction induces activation through a conformational change [10]. Once activated, ROCK orchestrates cytoskeletal remodelling and cell behaviour by phosphorylating multiple downstream substrates involved in contraction, adhesion, migration, proliferation, and apoptosis—key processes in corneal physiology and wound healing [10,11].

There are two isoforms of ROCK—ROCK 1 and ROCK 2—which share approximately 65% overall sequence identity and 92% homology in their kinase domains [12]. While their functions overlap, they differ in tissue distribution and localization. ROCK 1 is more prominent in mesenchymal and immune tissues, whereas ROCK 2 is enriched in the nervous system, cardiovascular tissues, and ocular structures, including the cornea [11,13].

**Figure 1:**
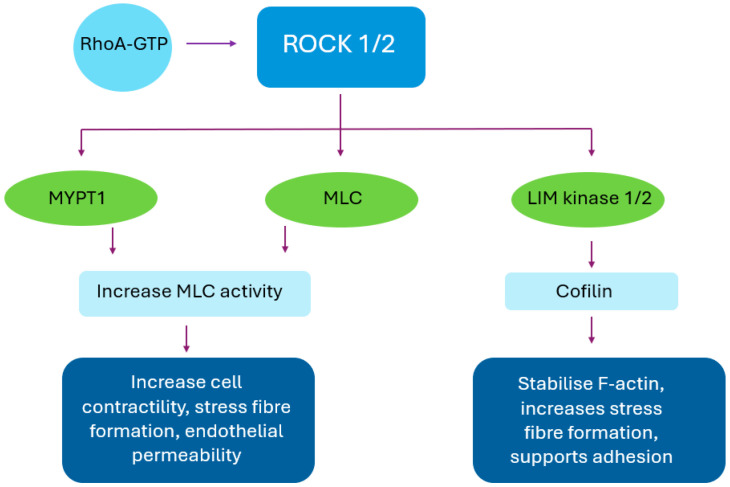
RhoA/ROCK signalling pathway in the cornea. This is a schematic representation of key downstream targets of RhoA-activated ROCK1/2 and their roles in modulating actin cytoskeleton organisation, cell contractility, and adhesion. In the corneal context, these processes influence barrier integrity, cell–matrix interactions, and wound healing, essential for maintaining transparency and responding to injury. ROCK: Rho-associated coiled-coil containing protein. MYPT1: myosin phosphatase target subunit 1. MLC: myosin light chain.

Functionally, both isoforms drive cytoskeletal remodelling by phosphorylating myosin light chain (MLC) and inhibiting myosin phosphatase via phosphorylation of myosin phosphatase target subunit 1 (MYPT1) [10]. This enhances MLC activity, resulting in increased cell contractility, stress fibre formation, and focal adhesion assembly [10,11]. ROCK also activates LIM kinase, which phosphorylates and inactivates cofilin, preventing actin filament disassembly and reinforcing cytoskeletal stiffness [10,11]. Collectively, these actions increase cell tension and adhesion while limiting cell motility under normal conditions (Figure 1).

In the corneal epithelium, ROCK is activated in response to growth factors or injury, coordinating the cytoskeletal changes required for sheet migration and re-epithelialization [2]. Inhibition of ROCK reduces actomyosin tension, promotes cell spreading, and enhances wound closure [2,14,15]. For instance, ROCK inhibition with Y-27632 accelerated epithelial wound closure by enhancing corneal epithelial cell migration and adhesion, without promoting proliferation [2].

In the corneal stroma, ROCK is a central mediator of transforming growth factor beta (TGF-β)–induced differentiation of keratocytes into alpha smooth muscle actin (α-SMA) positive myofibroblasts, a key step in fibrotic scar formation [16,17]. ROCK activity supports this phenotypic switch by increasing cellular contractility and driving expression of profibrotic genes such as collagen I, fibronectin, and α-SMA [13,17]. Yamamoto et al. demonstrated that Y-27632 in rabbit corneal keratocytes almost abolished TGF-β1-induced myofibroblast conversion, reducing α-SMA-positive cells from 4% to 0.3% [16]. Similarly, topical administration of Y-27632 after keratectomy dramatically reduced stromal α-SMA expression and altered collagen deposition toward an embryonic-like pattern [16]. Thus, ROCKI may limit scarring by blocking the fibroproliferative program.

In the corneal endothelium, ROCK signalling is essential for maintaining barrier integrity and cellular architecture because it stabilises the actin cytoskeleton and junctional/pump complexes such as zonula occludens-1 (ZO-1) and sodium–potassium adenosine triphosphatase (Na^+^/K^+^-ATPase) [18]. Under oxidative or inflammatory stress, the pathway is over-activated and excessive actomyosin contractility leads to cell contraction, junctional breakdown and caspase-3–mediated apoptosis [19]. Pharmacological ROCK blockade (Y-27632, Y-39983, ripasudil) can reverse these changes: it relaxes the cytoskeleton, promotes cell spreading, triggers a cyclin-D–dependent proliferation, and increases endothelial adherence to extracellular matrix or Descemet’s membrane [20,21]. In addition, recent work shows that ROCKI directly mitigate oxidative damage by lowering intracellular reactive-oxygen-species (ROS) levels, preserving mitochondrial membrane potential in endothelial cultures, and reducing oxidative-stress-induced apoptosis in both endothelial and epithelial models [22,23].

These cytoprotective and pro-migratory effects have been exploited clinically. After Descemet stripping only (DSO) or intracameral injection of cultured endothelial cells, adjunctive ROCKI accelerates endothelial migration and repopulation of the posterior corneal surface, restoring corneal clarity more rapidly [6,8,21,22]. In FECD tissue, ROCKI exposure with ripasudil up-regulated transcripts and proteins related to cell-cycle progression, adhesion, migration and pump function, promoting functional restoration without inducing fibrotic change [7]. Complementary research further demonstrated that ripasudil stimulates Rac1, up-regulates Snail and vimentin, and markedly enhances FECD-cell motility in vitro and in an ex vivo DSO model, supporting the hypothesis that ROCKI may convert normally quiescent corneal endothelium to a reparative phenotype [23].

**Figure 2:**
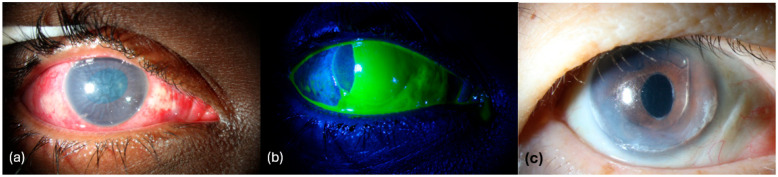
Clinical images of patients who may benefit from ROCKI use—Images (**a**,**b**) are of a patient who experienced a severe chemical injury with the presence of a large corneal epithelial defect with stromal oedema, and significant conjunctival and limbal involvement. Image (**c**) is of a patient who underwent complicated cataract surgery with subsequent insertion of an anterior chamber intraocular lens. The postoperative recovery was complicated by development of pseudophakic bullous keratopathy.

ROCKI also exerts anti-inflammatory and anti-angiogenic effects in the cornea. ROCK activity promotes nuclear factor kappa B (NF-κB) mediated cytokine production and immune cell recruitment, while ROCKI reduces inflammation and fibrosis [24,25]. Their antioxidative action further dampens ROS-driven inflammatory cascades, providing a complementary mechanism for tissue protection [26,27]. In ocular surgery models, topical ROCKI suppresses fibroblast proliferation and myofibroblast differentiation, resulting in reduced stromal scarring [16]. In a murine model of corneal transplantation, ROCKI has been shown to reduce graft rejection by promoting regulatory T cell expansion and suppressing T-helper 17 (Th17) responses [28,29]. ROCK blockade has also been shown to inhibit pathologic neovascularization [30]. In murine corneas, Y-27632 curbed Sonic-Hedgehog–driven vessel ingrowth, while topical fasudil markedly reduces alkali-burn–induced corneal neovascularization [30,31] (Figure 2).

In summary, ROCK acts as a regulator of cytoskeletal dynamics, proliferation, apoptosis, inflammation and fibrosis in corneal cells. Blocking Rho–ROCK signalling relaxes the cytoskeleton, enabling endothelial migration/proliferation, and dampens pathologic wound healing such as inflammation and neovascularization in the cornea, as summarised in Table 1. These benefits are now translated into adjunctive therapies for DSO, cell injection, and post-surgical wound healing.

## 4. Pharmacological Properties

Most ROCKI available at present are non-selective and inhibit both isoforms (ROCK 1 and 2), though newer agents with isoform selectivity are under development [32,33,34]. ROCKI are typically low-molecular-weight small molecules designed for effective tissue penetration [32]. These agents were originally developed for glaucoma therapy due to their ability to lower intraocular pressure (IOP) via trabecular meshwork relaxation, but their pharmacologic profiles also support emerging corneal applications [32], which can be summarised in Table 2 below.

Several ROCKI have been developed with varying degrees of selectivity and pharmacokinetic profiles. The prototype pan-ROCKI Y-27632 is widely used in pre-clinical studies; it works as an ATP-competitive inhibitor that reversibly binds to the ATP pocket of ROCK, displacing ATP and preventing substrate phosphorylation [35,36]. This pyridine-substituted small molecule inhibits ROCK 1 and 2 at nanomolar levels, but exhibits off-target activity at higher concentrations and begins to block other AGC-family kinases—such as protein kinase C related protein kinase 2 and protein kinase A [35,37,38]. Fasudil and its active metabolite hydroxyfasudil, another non-selective ROCKI, has been approved in Japan for intravenous use in cerebral vasospasm and is currently evaluated intravitreally for retinal edema and other ophthalmic indications [36,39]. Like Y-27632, fasudil binds competitively at the ATP site of ROCK [36]. Because both Y-27632 and fasudil inhibit additional serine/threonine kinases at higher doses, careful dose selection is essential to minimize off-target effects.

In clinical practice, established agents are ripasudil (Glanatec^®^, 0.4% solution) and netarsudil (Rhopressa^®^, 0.02% solution). Although label strengths differ by a factor of 20, this does not mean ripasudil is intrinsically more potent; the higher concentration compensates for formulation and ocular residence-time differences [40,41,42,43,44]. Ripasudil is approved for glaucoma in Japan and China, and is typically administered twice daily [32]. Netarsudil, approved in the United States and European countries, is dosed once daily and exhibits dual activity as both a ROCKI and a norepinephrine transporter (NET) inhibitor [45]. While initially developed for intraocular pressure reduction, both agents are being investigated for their utility in corneal wound healing, endothelial regeneration, and post-surgical recovery [18,46,47]. Several additional ROCKI are under development. SNJ-1656 (Y-39983), a more potent derivative of Y-27632, completed phase II trials for lowering IOP in open-angle glaucoma [48,49]. It is also frequently employed in vitro and in animal models to probe ROCK signalling in ocular tissues [33]. Beyond pan-ROCK agents, newer compounds with isoform selectivity, such as topical ITRI-E-212 and NRL-1049, which favour ROCK 2 over ROCK, are investigated to refine tissue targeting and reduce off-target effects [50,51].

From a pharmacokinetic standpoint, topically instilled ROCKI achieved high anterior-segment concentrations while systemic exposure remains negligible due to limited corneal absorption and any excess drug is quickly lost through the nasolacrimal duct [40]. After netarsudil 0.02% was given once daily for eight days, no plasma samples contained parent drug above the 0.1 ng mL^−1^ limit of quantification, and only one metabolite sample reached 0.11 ng mL^−1^—evidence of negligible systemic exposure [41].

Ripasudil 0.4% shows a similar profile: autoradiography confirms rapid corneal uptake and aqueous-humour distribution in rabbits, and human studies report a systemic half-life of just 0.5–0.7 h with peak plasma levels ≤ 0.7 ng mL^−1^ [40,42]. Published data show that topical ripasudil 0.4% produces corneal effects lasting ~6 h, whereas netarsudil 0.02% demonstrates a corneal elimination half-life of ~13–14 h in rabbit studies; human ex vivo tissue studies report a shorter half-life of ~3 h [43,44]. Both agents are metabolised rapidly within the eye and are cleared via nasolacrimal drainage, and because tear-film/aqueous humour protein levels are very low, neither drug exhibits meaningful protein binding in these compartments [45,52,53]. Consequently, therapeutic levels persist for a short period of time.

This short duration of action of current ROCKI has prompted development of sustained-release formulations. These include nanocarriers, in situ gelling systems, and intracameral delivery devices designed to enhance ocular retention and reduce dosing frequency [32,54,55,56]. Such innovations may broaden the clinical utility of ROCKI in chronic corneal conditions and surgical recovery.

## 5. Safety Profile

Ripasudil and netarsudil have been reported to be generally safe for corneal and ocular use, with mostly localized ocular side effects and minimal systemic impact [5]. Clinical trials and post-marketing studies indicate very few drug-related serious ocular adverse events, and systemic events are rare and comparable to those with standard treatments [57]. The safety profile of these agents is summarized below, highlighting common adverse effects, their frequency and severity, and relevant precautions:

### 5.1. Conjunctival Hyperaemia

This is the most common adverse effect of ROCKI. A majority of patients experience some degree of conjunctival redness (e.g., ~50–55% with netarsudil and 60–65% with ripasudil) [57,58]. The hyperaemia is typically reported as mild and transient, often resolving shortly following instillation [59]. Notably, hyperaemia tends to decrease over time with continued use [45]. Despite its frequency, conjunctival injection rarely leads to discontinuation [60].

### 5.2. Blepharitis and Periocular Irritation

Blepharitis, sometimes with symptomatic itching and eyelid erythema, is a significant issue particularly for ripasudil. Over 12 months, blepharitis was reported to have developed in approximately 25% of ripasudil-treated patients and is consistently reported as the most frequent cause of treatment discontinuation, accounting for 21% and 34.6% of patients discontinuing within the first and second year respectively [61]. Extended monitoring over two years found blepharitis to be the most common adverse event (~8% incidence in a large cohort of 3374 patients) with most cases reported as mild and resolve spontaneously upon drug cessation, usually within four weeks [61,62]. Patients with atopy or prior topical drug allergies are at higher risk for ripasudil-induced blepharitis [61,62]. In contrast, netarsudil rarely causes blepharitis; this difference in lid tolerability between agents is notable, though the underlying reason remains unclear [63].

### 5.3. Corneal Verticillata (Vortex Keratopathy)

ROCKI can induce characteristic corneal epithelial deposits. Netarsudil in particular has been associated with cornea verticillata in 20% of patients [45,64]. These appear as faint golden-brown whorl-like patterns in the corneal epithelium and typically emerge after several weeks of therapy [57]. These corneal deposits are benign and neither cause loss of visual acuity or lasting corneal damage, disappearing within weeks of discontinuing medication [64,65]. Therefore, it usually does not necessitate stopping treatment unless patients experience severe manifestations which occurs in ~4% of cases [64]. It is postulated that netarsudil possesses chemical characteristics similar to cationic amphiphilic drugs, such as amiodarone, known to induce phospholipidosis, which leads to intracellular phospholipid accumulation and corneal verticillata development [66]. In contrast, ripasudil lack these structural features and has not been documented to cause corneal verticillata [66,67].

### 5.4. Conjunctival Haemorrhages

Small, asymptomatic subconjunctival haemorrhages occur as a result of conjunctival vessel dilation arising from ROCKI. In netarsudil-treated eyes, these microhaemorrhages are frequently reported (often ranked after hyperaemia and cornea verticillata, around 17% of cases) [64]. They typically present as tiny petechial red spots on the ocular surface, often around the limbus [45]. Crucially, these haemorrhages are self-limiting and benign, and resolve with continued use [64]. These haemorrhages occur less frequently with ripasudil as reported through phase III trials and in post-marketing use [57,68].

### 5.5. Honeycomb Keratopathy

Both ripasudil and netarsudil have been linked to the development of transient corneal epithelial oedema with a distinctive honeycomb or reticular pattern [69,70,71]. This phenomenon, also described as reticular epithelial oedema or “honeycomb” keratopathy, has occurred in patients with compromised corneas. Risk factors include prior corneal transplantation, corneal decompensation, and recent glaucoma procedures, particularly cyclophotocoagulation [72,73,74,75]. While the exact pathogenesis remains unknown [76], it was postulated that pre-existing stromal oedema may shift into the corneal epithelium through ROCKI mediated increase in tight junction permeability.

The nature and time course of honeycomb epithelial oedema may vary between ROCKI, with netarsudil often associated with earlier onset [5]. In most reported cases, this was reversible after stopping or reducing frequency of ROCKI administration, typically resolving over days to weeks [72,73]. One case with ripasudil in a post-descemet stripping automated endothelial keratoplasty (DSAEK) patient required a repeat corneal transplant to restore vision has been reported [77]. However, the reported case had pre-existing corneal decompensation prior to commencing ripadusil and cessation of drug was therefore not considered. Nevertheless, with ROCKI gaining popularity as a treatment for corneal oedema, clinicians should monitor for development of honeycomb keratopathy and associated complications from bullae ruptures, especially in eyes at risk.

### 5.6. Other Mild Ocular Effects

A variety of transient, mild ocular symptoms have been documented with ROCKI instillation. These include pain or stinging, mild conjunctival irritation, blurred vision immediately following administration, and corneal staining [45,78,79,80]. These are generally mild and short-lived. Occasional headaches have been reported in a minority of patients on netarsudil, but no consistent systemic neurological effects are observed [81].

### 5.7. Serious Adverse Events

Serious adverse events with netarsudil or ripasudil are rare [64,67,81]. Large phase III trials reported no clinically relevant changes in blood pressure, heart rate, or other systemic parameters, and the overall incidence of non-ocular serious events with netarsudil (~3.3%) matches that of timolol (~3.2%) [64,82]. Reported serious adverse events include coronary artery disease, myocardial infarction and prostate cancer [64]. Post-marketing pharmacovigilance has likewise not revealed any systemic safety concerns unique to these drugs [67,83]. However, a recent analysis of FDA adverse event reports has demonstrated several cases of systemic hypersensitivity reactions temporally associated with netarsudil [81]. Although such reactions are exceedingly infrequent, prescribers ought to remain aware of the potential for idiosyncratic allergy.

### 5.8. Contraindications and Precautions

There are no absolute contraindications for topical netarsudil or ripasudil aside from known hypersensitivity to the drug or its components [45]. Patients with a history of severe allergic reactions to ocular medications should be observed carefully if started on ripasudil, given the noted association of atopy with ROCKI-related blepharitis [62]. If significant allergic conjunctivitis or blepharitis develops, therapy may need to be discontinued. Additionally, while not contraindicated, the use of ROCKI in children or pregnant women has not been extensively studied; only rare pediatric cases (e.g., transient corneal changes in a child on netarsudil) have been reported, so these populations warrant cautious use and close monitoring [84,85].

Ripasudil and netarsudil have a demonstrable favourable safety profile. This safety profile, combined with their therapeutic benefits, supports continued and expanding indications of applying ROCKI to glaucoma and a range of corneal disorders, provided that patients are appropriately counselled and reviewed for the known side effects.

## 6. Corneal Oedema

Corneal oedema arises when endothelial pumps fail to function effectively. ROCKI have reported efficacy across a range of conditions that can result in corneal oedema, often by promoting residual endothelial cells to repopulate denuded areas. We address three key conditions:

### 6.1. Fuchs’ Endothelial Corneal Dystrophy

#### 6.1.1. Pathophysiology

FECD is a non-inflammatory, degenerative dystrophy of the central corneal endothelium characterized by progressive endothelial cell loss, polymegethism and pleomorphism, and formation of extracellular matrix excrescences (guttae) on Descemet’s membrane, leading to chronic stromal oedema, glare and visual decline [86]. Medical therapy such as hyperosmotic drops may temporarily improve symptoms but do not halt disease progression [87]. In advanced FECD, endothelial keratoplasty (EK) such as descemet membrane endothelial keratoplasty (DMEK) or DSAEK is required to restore vision [86,87].

#### 6.1.2. Mechanistic Rationale for ROCKI

CECs possess limited in vivo proliferative capacity under normal conditions [88]. Nonetheless, primary human CECs can be expanded ex vivo with a dual-media culture system that preserves their phenotype [89]. Exposure of these propagated cells to ROCKI Y-27632 further enhances proliferation, barrier integrity and pump-function gene expression [90]. ROCKI can also induce cell proliferation and migration [20,21,87]. In FECD, stimulating peripheral healthy CECs migration centrally can clear corneal edema. Schlötzer-Schrehardt et al. reported that application of a single topical dose of ripasudil on excised human FECD tissue resulted in significant upregulation of cell-cycle, adhesion, migration, barrier and pump genes/proteins [7]. In effect, ROCKI reactivates regenerative programs in dysfunctional endothelium. DSO selectively removes the central diseased Descemet membrane and endothelium in the treatment of FECD [91]. It relies on peripheral CEC migration to repopulate the resultant central defect with topical ROCKI accelerating this process [92,93].

#### 6.1.3. Preclinical Evidence

In vitro and ex vivo studies confirm these concepts. Ex vivo human FECD tissue exposed to ripasudil demonstrated activated expression of genes and proteins related to proliferation, cell–matrix adhesion and migration, as well as endothelial barrier and pump function for up to 72 h [7]. Mesenchymal (fibrotic) markers were also suppressed [7]. In vitro scratch-wound assays using immortalized normal and FECD CECs demonstrated that ripasudil significantly enhanced wound-closure rates and migration speeds compared to controls [23]. Predictive work has identified young donor age, an intact Descemet membrane and Y-27632 supplementation as key determinants of HCEC migratory capacity [94]. Other animal studies have also provided evidence that ROCKI promotes endothelial healing: topical Y-27632 applied after transcorneal freezing in cynomolgus monkeys accelerated endothelial wound healing, restoring cell densities to ~3000 cells/mm^2^ compared to ~1500 cells/mm^2^ in untreated controls, with recovered ZO-1 and Na^+^/K^+^-ATPase expression [18,95].

#### 6.1.4. Clinical Studies

Several pilot studies and case series have reported the efficacy of ROCKI in FECD. Lindstrom et al. randomized 40 patients with FECD to netarsudil once daily or twice daily for eight weeks [96]. Netarsudil reduced central corneal thickness (CCT) by 28.4 µm (in the once daily group) and 20.1 µm (in the twice daily group) [96]. 12.5% of eyes achieved complete resolution of corneal oedema and improvements in visual acuity (>10 letters) occurred in 25% of treated eyes [96]. These improvements persisted with no significant differences between dosing regimens at any time point [96]. A double-masked pilot trial by Price et al. randomized 29 FECD patients to netarsudil 0.02% once daily or placebo for 3 months; with treated eyes showing mean CCT reductions of 20 µm at one month and 26 µm at three months, plus a 1.6-line scotopic visual acuity gain at three months, though visual disability scores were unchanged and one patient withdrew due to glare [97]. A clinical series of eight corneal endothelial dysfunction patients treated with topical Y-27632 administered six times daily after transcorneal freezing reported improvement in corneal oedema [18]. Building on that protocol, Tomioka et al. have now published a 12-year follow-up of an early-stage FECD eye treated with 2 mm trans-corneal freezing and one week of Y-27632 four times daily: corneal transparency returned within two weeks and remained clear at 12 years, with a central endothelial-cell density of 1294 cells mm^−2^ and an annual cell-loss rate of just 1.1% [98].

In practice, many investigators have evaluated DSO combined with topical ROCKI in FECD. Moloney et al. treated 23 eyes of 23 patients with DSO followed by ripasudil 0.4% six times daily until corneal clearance was achieved; 22 eyes cleared at a mean of 4.1 weeks, with mean uncorrected visual acuity and best-spectacle-corrected visual acuity improvements of 0.20 and 0.156 LogMAR respectively [8]. Although nine eyes experienced relapse on cessation, this resolved upon restarting treatment [8]. In a prospective, non-randomized comparison, Macsai and Shiloach enrolled 18 patients undergoing DSO (9 received ripasudil 0.4% four times daily for two months post-DSO; nine were observed without drug); ripasudil-treated eyes regained clarity faster (mean 4.6 vs. 6.5 weeks, *p* < 0.01) and maintained peripheral endothelial cell density at 12 months, whereas controls experienced a 10% density decline [93]. Davies et al. performed a pilot study in ten FECD patients (20 eyes) undergoing DSO with cataract surgery; immediate administration of netarsudil following DSO halved clearance time compared to delayed use (‘rescue’ with netarsudil in persistent corneal oedema), and the average time for cornea clearance was 4.6 ± 1.7 and 8 ± 1.9 weeks respectively (*p* < 0.01), with improved postoperative endothelial cell counts (*p* = 0.05) [92].

Two Phase III trials are underway; DETECT II (NCT05275972) is a multicenter, masked RCT of 60 FECD patients randomized to DSO and ripasudil 0.4% versus DMEK, with 12-month BCVA as the primary endpoint [99], while K-321 (NCT05795699) is a double-masked, placebo-controlled study of ripasudil post-DSO in ~100 patients (12-week dosing, 38-week follow-up) [100].

#### 6.1.5. Summary and Limitations

These data suggest ROCKI can awaken regenerative potential in FECD endothelium, especially when combined with central guttae removal (Table 3). However, existing evidence arises from small, largely non-randomized cohorts, with most responders experiencing mild-to-moderate FECD. Severely decompensated corneas are less likely to improve without corneal transplantation [101]. Upregulation of transient endothelial-mesenchymal markers has been observed in vitro studies, raising questions about phenotypic stability, though ex vivo FECD tissue showed overall suppression of fibrosis markers [7,23]. Clinically, repopulation after DSO and ROCKI is better in eyes with a larger peripheral endothelial cell reservoir, reflecting higher baseline cell counts, implying that older patients (who have lower cell densities) may have reduced in vivo regenerative capacity [8,93]. However, data on the long-term outcomes of DSO and ROCKI (beyond a year)—apart from a single 12-year case report after trans-corneal freezing and short-term Y-27632 therapy—remains limited [98]. ROCKI represent a promising medical therapy for early FECD, but larger, controlled trials with longer follow up will be useful to define optimal regimens and ideal patient selection.

### 6.2. Pseudophakic Bullous Keratopathy

#### 6.2.1. Pathophysiology

PBK occurs due to corneal endothelial trauma which leads to widespread endothelial cell loss [102]. Although PBK shares its final common pathway of endothelial pump failure with FECD, its onset is typically more acute [86,102].

#### 6.2.2. Mechanistic Rationale for ROCKI

Analogous to FECD, ROCKI may enhance residual endothelial cell migration and improve pump function to reduce oedema [3]. In post-surgical PBK, inflammation and oxidative stress further contribute to cell loss; ROCKI’s anti-apoptotic effects could protect surviving endothelium [103]. Furthermore, ROCKI relaxes trabecular meshwork cells, increases conventional aqueous outflow through Schlemm’s canal, and lowers episcleral venous pressure, which may mitigate transient IOP elevations that can exacerbate corneal oedema post-surgery [32].

#### 6.2.3. Preclinical Evidence

Okumura et al.’s primate cryoinjury study mentioned above is relevant to this condition as it models endothelial failure similar to PBK [18]. Accelerated healing and cell density gain with Y-27632 supports use of ROCKI in pseudophakic oedema to promote regeneration and endothelial pump recovery in PBK.

#### 6.2.4. Clinical Studies

In an uncontrolled, single-group trial by Kinoshita et al., 11 eyes with bullous keratopathy received intracameral injection of 1 × 10^6^ cultured human CEC supplemented with the ROCK-i Y-27632 [6]. At 24 weeks, all 11 eyes (100%) achieved restoration of corneal transparency and attained CEC densities ranging from 947 to 2833 cells/mm^2^ [6]. This highlighted the role of ROCKI in facilitating the engraftment and function of transplanted CECs in bullous keratopathy.

In a retrospective case series, three PBK eyes were treated with topical ripasudil 0.4% four times daily [104]. Two achieved partial to complete oedema resolution and improved visual acuity within 2–4 weeks, while the third experienced initial clearing on ripasudil but redeveloped progressive oedema after treatment cessation and ultimately required an EK [104]. In another case series, three advanced PBK patients were treated with ripasudil 0.4% thrice daily for three to eleven months. [105]. All patients demonstrated reductions in CCT, improved best-corrected visual acuity, and stable or modestly increased endothelial cell counts [105]. Conversely, a case report described netarsudil 0.02% once daily failing to clear PBK-associated oedema after 12 weeks [106]. A Phase I clinical trial (NCT06960629) by University Hospital Dubrava is currently recruiting to evaluate a combined netarsudil/latanoprost formulation for PBK and related corneal endothelial dysfunction [107].

#### 6.2.5. Summary and Limitations

The evidence for ROCKI in PBK is limited to anecdotal reports and small case series [104,105]. ROCKI show promise as adjuncts to cell therapy and as topical treatment in early PBK, but no large trials presently exist [6]. In established bullous edema, medical management alone is typically insufficient, and most patients ultimately require EK [102]. Topical ROCKI may however be useful as adjunctive treatment to stabilize corneas awaiting surgery. However, PBK patients—often older with low endothelial reserves—may derive only modest regenerative benefit.

### 6.3. Iridocorneal Endothelial Syndrome

#### 6.3.1. Pathophysiology

Iridocorneal Endothelial Syndrome (ICE) encompasses a spectrum of unilateral disorders—including Chandler syndrome, Cogan-Reese syndrome, and essential iris atrophy—characterized by abnormal proliferation and migration of CEC onto the iris and anterior chamber angle [108].

#### 6.3.2. Mechanistic Rationale for ROCKI

Since ICE syndrome is fundamentally a disorder of corneal endothelial dysfunction, ROCKI have been proposed to be useful in promoting corneal endothelial function [109]. Furthermore, as secondary angle-closure glaucoma arises from proliferation and migration of CEC to the angles; commercially available ROCKI like netarsudil lowers IOP by increasing aqueous drainage through episcleral venous dilation and expansion of cribiform meshwork [32,110,111].

#### 6.3.3. Preclinical Evidence

There are currently no disease-specific models of ICE, limiting in vivo and in vitro insights into how ROCK inhibitors affect its endothelial pathology.

#### 6.3.4. Clinical Studies

Evidence for ROCKI in ICE is extremely limited. Davies et al. reported a case of an 80-year-old woman with corneal oedema on a background of ICE syndrome who experienced significant regression of stromal oedema and improved best-corrected visual acuity after four weeks of once-daily netarsudil 0.02% eye drops [106]. Beyond anecdotal evidence, no prospective or controlled trials have been undertaken.

#### 6.3.5. Summary and Limitations

Given the limited evidence, the role of ROCK inhibitors in ICE remains speculative. Their safety and efficacy in this context are unclear, and further studies are needed to define their potential use.

## 7. Corneal Neovascularization

### 7.1. Pathophysiology

Pathological corneal neovascularization refers to ingrowth of new blood vessels and lymphatics from the limbal vascular plexus into the avascular cornea, usually in response to inflammation, infection, or hypoxia [112]. Ingrowth of fragile, leaky vessels scatters light and provokes stromal inflammation and edema [113]. Vessels within a corneal graft bed is also a well-established risk factor for corneal allograft rejection [114,115]. Key molecular drivers include vascular endothelial growth factor (VEGF) and other pro-angiogenic cytokines, which upregulate matrix metalloproteinases to allow endothelial cell migration and tube formation [112,116].

### 7.2. Mechanistic Rationale for ROCKI

ROCK 1 and 2 drive actomyosin contractility and cytoskeletal remodelling essential for endothelial and smooth muscle function [117]. In vascular endothelial cells, ROCK signalling is essential for VEGF-mediated processes including endothelial cell migration, survival, permeability, and capillary-like tube formation; pharmacologic inhibition with the ROCKI Y-27632 disrupts VEGF-induced tube formation in vitro [118,119]. In smooth muscle, ROCK phosphorylates MLC and inhibits myosin phosphatase, increasing Ca^2+^ sensitivity and contractility to regulate vessel tone [117,120,121]. In addition, RhoA/ROCK signalling also regulates lymphatic endothelial cell (LEC) junction integrity: inhibition with Y-27632 induces “zipper-like” junctions in LECs, altering permeability and LEC sprouting—suggesting potential anti-lymphangiogenic effects [122].

### 7.3. Preclinical Evidence

A growing body of in vitro and in vivo studies supports the anti-angiogenic properties of ROCKI. In cultured endothelial cells, treatment with Y-27632 significantly impairs VEGF-induced migration and capillary-like tube formation [118]. An animal study on alkali burn injury in murine corneas with topical fasudil showed significant reduction in neovascularization compared to corneas treated with phosphate-buffered saline, decreased inflammatory cell infiltration and reactive oxygen species, downregulated VEGF, TNF-α, MMP-8/9 mRNA, and upregulated heme oxygenase-1, while accelerating corneal epithelial healing [30]. Similar effects in reducing corneal neovascularization have been reported by a study using the mouse corneal micropocket assay [123]. AMA0526, a novel ROCKI, improved corneal clarity, and significantly reduced the area of corneal neovascularization by 37%, type III collagen deposition by 27%, inflammatory cell infiltration by 26%, and angiogenesis by 47% [123]. Comparing AMA0526 against dexamethasone, this ROCKI demonstrated similar effects with possible greater antifibrotic properties as demonstrated by histological evidence of reduced collagen deposition [123].

### 7.4. Clinical Studies

To date, no clinical trials have tested ROCKI specifically for corneal neovascularization.

### 7.5. Summary and Limitations

Preclinical models consistently demonstrate that ROCKI (Y-27632, fasudil, AMA0526) suppresses corneal neovascularization by blocking endothelial sprouting, reducing inflammation, and promoting epithelial repair. Although pre-clinical studies show that ROCKI suppress corneal neovascularisation and the fibroblast-to-myofibroblast transition, no prospective human trial has yet confirmed an anti-scarring or anti-angiogenic benefit, and clinical experience remains anecdotal [29]. However, these findings support exploration of ROCKI, alone or in combination with anti-VEGF/steroids, for disorders with corneal neovascularization, particularly in high-risk graft and burn-related cases. While ROCKIs are generally less potent IOP-lowering agents than first-line prostaglandins, they may outperform conventional drops in conditions dominated by trabecular outflow resistance—such as pigment-dispersion glaucoma, inflammatory (uveitic) pressure spikes, or post-vitrectomy ocular hypertension [124]. Potential niche indications include eyes with vascularized stromal scars following keratectomy or herpes simplex virus (HSV) stromal keratitis, where fibrosis and neovascularization are prominent. Clinical translation awaits dedicated studies to assess safety, optimal dosing, and efficacy.

## 8. Post-Cataract Surgery Corneal Healing

### 8.1. Pathophysiology

Cataract surgery (phacoemulsification) inevitably results in loss of CEC, which can potentially cause stromal edema due to pump and barrier dysfunction [125,126]. Patients with low preoperative endothelial cell density (e.g., <1000 cells/mm^2^) are at risk for corneal decompensation [126]. The acute surgical insult further triggers a postoperative inflammatory response (increased levels of TNF-α, IL-6, IL-8, and other cytokines) and reactive oxygen species production, inducing endothelial apoptosis and autophagy [127,128,129].

### 8.2. Mechanistic Rationale for ROCKI

ROCKI may protect CEC from surgical stress and enhance recovery. ROCKI suppresses pro-apoptotic signalling downstream of TNF-α and oxidative stress [130], promoting endothelial survival. It also stabilises endothelial cytoskeleton after stress, preserving cell shape and facilitating resealing of disrupted junctions [109]. Furthermore, barrier integrity is enhanced, upregulating tight-junction and adherens-junction proteins (e.g., ZO-1, occludin), thereby reducing paracellular leak and accelerating stromal deturgescence [10]. Additionally, ROCKI possess anti-inflammatory effects that may mitigate postoperative inflammation [131].

### 8.3. Preclinical Evidence

Experimental evidence in corneal healing after cataract surgery is limited, but related models support a protective effect. Y-27632 and related ROCKI accelerate endothelial wound healing, suppress fibrosis, and boost cell proliferation and adhesion in animal and in vitro studies [3]. Topical application has been reported to restore a high-density functional endothelial monolayer in rabbit and primate models [21,132].

### 8.4. Clinical Studies

Two recent human trials have tested ripasudil in the postoperative setting. Fujimoto et al. conducted a retrospective observational study in 16 eyes of patients diagnosed with glaucoma with low preoperative endothelial cell density (<1500 cells/mm^2^) who received 0.4% ripasudil eye drops twice daily after phacoemulsification, compared to 17 control eyes without ripasudil [133]. At one week postoperatively, the ripasudil group showed a significantly reduced increase in corneal thickness (1.25% vs. 5.97%; *p* = 0.0037), and at 90–120 days, endothelial cell loss was markedly lower in the ripasudil group (4.5% loss vs. 14.1% loss; *p* = 0.0003) [133]. Similarly, in a prospective, non-randomized comparative study of 43 eyes undergoing uneventful phacoemulsification, patients receiving ripasudil 0.4% three times daily for five days (23 eyes) were compared to controls (21 eyes) [134]. At one year, the ripasudil group experienced significantly less endothelial cell loss (4.5% vs. 12.8% in controls; *p* = 0.001) [134]. These results suggest that topical ripasudil may assist in preserving corneal endothelial integrity and after cataract surgery.

### 8.5. Summary and Limitations

Topical ripasudil has been shown to reduce endothelial cell loss after phacoemulsification but has only been studied in small, non-randomized cohorts. It is also unclear whether other ROCKI (e.g., netarsudil) confer similar effects. Moreover, ROCKI does not completely prevent cell loss, and although reported toxicity is minimal, optimal dosing regimens and safety profiles require further clarification. Adequately powered randomized trials with extended follow-up will be useful in defining patient selection, dosing strategies, and long-term safety before ROCKI can be adopted as a standard adjunct to cataract surgery.

## 9. Corneal Fibrosis and Wound Healing

### 9.1. Pathophysiology

Corneal fibrosis (haze or scarring) results from abnormal wound healing after injury, such as abrasion, keratitis, or refractive/surgical trauma, leading to stromal opacification [135]. This leads to TGF-β production which drives quiescent keratocytes to differentiate into α-SMA positive myofibroblasts; these cells generate contractile stress fibres, deposit a disorganized extracellular matrix, and result in stromal contraction, increasing light scattering and reducing transparency [136,137,138]. Normally, the intact epithelium and basement membrane restricts stromal exposure to pro-fibrotic cytokines; once disrupted, TGF-β enters the stroma and triggers fibroproliferation [135].

### 9.2. Mechanistic Rationale for ROCKI

Since ROCK regulates actomyosin contraction and stress fibre formation [10], inhibition of ROCK can prevent myofibroblast contraction, downregulate fibrotic signalling pathways, and demonstrates broad anti-fibrotic effects in models of pulmonary and renal fibrosis [139,140]. In the cornea, blocking ROCK (e.g., with Y-27632) during the healing phase suppresses α-SMA expression and limits TGF-β1–mediated stress fibre formation and extracellular-matrix remodelling, which assists with preservation of corneal transparency by blocking the keratocyte-to-myofibroblast switch and favouring a more regenerative extracellular matrix organization [16,136,141].

### 9.3. Preclinical Evidence

There are few studies exploring the role of ROCKI in corneal fibrosis, but the available data are striking. Koizumi et al. first showed in vitro that rabbit corneal keratocytes stimulated with 3 ng/mL TGF-β1 converted into α-SMA^+^ myofibroblasts at a rate of 4%, whereas pre-treatment with Y-27632 reduced this to just 0.3% (*p* < 0.01) [16]. In a rabbit lamellar keratectomy model in the same study, exposure to twice-daily topical Y-27632 for 21 days markedly suppressed central stromal α-SMA expression, attenuated collagen III deposition, and promoted bundles of aligned, embryonic-like collagen fibrils in the anterior stroma, resulting in reduced stromal haze [16]. Beyond the cornea, systemic fasudil administration in mice prevented postoperative capsular contraction after cataract surgery by blocking lens epithelial–myofibroblast differentiation [142]. Dai et al. further demonstrated that Y-27632 decreased fibronectin, collagen I, and α-SMA expression in primary human pterygium fibroblasts, underscoring ROCKI’s broad antifibrotic potential in ocular tissues [143].

### 9.4. Clinical Studies

To date, no clinical trials have specifically evaluated ROCKI in the treatment of corneal haze or stromal scarring in humans. However, preclinical models suggest potential applications in clinical settings where development of corneal haze is likely (e.g., photorefractive keratectomy, chemical injury). In these contexts, prolonged exposure to anti-fibrotic agents during the stromal remodeling phase is often necessary to suppress myofibroblast formation and matrix disorganization. Given the short duration of action and limited corneal retention of current ROCK inhibitors, ongoing work is needed to explore optimized ophthalmic delivery systems that can sustain ROCKI release during the healing period and enhance their therapeutic efficacy in preventing haze [144].

### 9.5. Summary

Preclinical studies consistently show that ROCK is required for corneal myofibroblast formation and scarring, and Y-27632 can significantly reduce this transition. By blocking actomyosin-driven remodelling, ROCKI offers a strategy to promote regenerative healing and transparency preservation. Key limitations include the absence of human efficacy and safety data, uncertain optimal timing of administration, and potential impacts on normal keratocyte function. Nonetheless, these findings support further development of ROCKI as an anti-scarring adjunct in corneal surgery and injury management.

## 10. Post-Corneal Transplantation (Graft Survival and Immune Modulation)

### 10.1. Pathophysiology

Corneal transplantation, whether penetrating keratoplasty or EK, disrupts the corneal architecture, triggering robust wound-healing responses (cell migration, proliferation, extracellular matrix remodelling) and exposes donor antigens to host immunity [145,146]. Despite the immune privilege of the ocular environment, the most common cause of graft failure is T cell mediated allogeneic rejection targeting donor endothelial cells [147,148]. In “high-risk” recipients, characterized by preexisting stromal inflammation or vascularization, graft-bed inflammation and neovascular/lymphatic ingrowth raise rejection rates to around 50%, even with maximal corticosteroid and cyclosporine prophylaxis [148,149,150,151].

### 10.2. Mechanistic Rationale for ROCKI

ROCK is a key regulator of cytoskeletal dynamics and immune cell trafficking. It promotes endothelial–leukocyte adhesion via actomyosin contractility and adhesion-molecule clustering; inhibition of ROCK reduces leukocyte infiltration and downregulates angiogenic and lymphangiogenic factors such as VEGF, TNF-α, and matrix mellatoproteinase-2/9 [29]. ROCK is also necessary for chemokine-driven T-cell polarization and migration: the selective inhibitor Y-27632 markedly inhibits chemokine induced T-cell chemotaxis [152]. Moreover, ROCK signalling sustains Th1 and Th17 effector responses, whereas its inhibition with fasudil suppresses interferon gamma producing Th1 and IL-17 producing Th17 cells [152]. ROCKI can also skew immunity toward tolerance by enhancing STAT5-mediated expansion of regulatory T cells while suppressing STAT3-driven Th17 differentiation [28]. ROCKI also suppress neovascularization and fibroblast activation, limiting stromal scarring [29]. Collectively, these mechanisms suggest that topical ROCKI applied to corneal grafts could simultaneously dampen local immune attack, inhibit neovascularization, and accelerate epithelial wound closure.

### 10.3. Preclinical Evidence

Recent animal studies support these concepts. In a murine high-risk corneal allograft model, Inomata et al. applied topical ripasudil (0.4% and 2.0%) thrice daily and reported significantly prolonged graft survival, reduced opacity and corneal neovascularization scores, decreased CD45^+^ leukocyte infiltration and mRNA expression of VEGF, TNF-α, and other angiogenic/inflammatory factors, and accelerated epithelial wound closure compared to controls [29]. In a separate allogeneic mouse penetrating keratoplasty study, Li et al. treated recipients with Y-27632, which upregulated STAT5 phosphorylation and downregulated STAT3 in CD4^+^ T cells, increased the proportion of CD4^+^CD25^+^FoxP3^+^Helios^+^ regulatory T cells, reduced IL-17A^+^ Th17 cells and mature dendritic cell infiltration, and ultimately attenuated graft rejection [28]. Together, these preclinical findings demonstrate that ROCKI can effectively modulate both innate and adaptive immune responses, suppress angiogenesis, and enhance epithelial repair in corneal transplantation models.

### 10.4. Clinical Studies

Clinically, limited case series have applied ROCKI to post-keratoplasty edema. Davies et al. reported two cases of corneal edema in penetrating keratoplasty graft failure treated with netarsudil (one early graft failure and one chronic graft failure) [106]. Both cases achieved corneal clearance (two and four weeks, respectively) with topical netarsudil [106]. Similarly, Tseng and Feder described two cases of cornea edema from graft failure, one descemet stripping endothelial keratoplasty and one penetrating keratoplasty; topical ripasudil led to improved vision and graft clarity [104]. These anecdotes suggest ROCKI may assist in clearing oedema in patients experiencing graft failure.

Despite these encouraging reports, formal evidence is lacking. To date, there are no published randomized trials evaluating ROCKI as adjunct to immunosuppressants in human corneal transplantation. A Phase IV trial (CTRI/2024/06/069429) exploring ripasudil for graft survival has been registered although recruitment but has yet to commence [153]. Results from the DETECT-1 trial (NCT 38286566) which assessed the role of adjuvant topical ripasudil during ultra thin-DSAEK versus DMEK in reducing endothelial cell loss have yet to be released [154]. Empiric use of ripasudil in glaucoma patients with prior grafts suggests safety, but its impact on graft longevity remains unstudied in humans.

### 10.5. Summary and Limitations

Preclinical studies consistently show that ROCKI can enhance corneal graft survival by reducing inflammation, angiogenesis, and fibroproliferation while promoting re-epithelialization, as demonstrated with topical ripasudil and Y-27632 in murine allograft models. This evidence underpins a novel immunotherapeutic approach: using topical ROCKI as a steroid-sparing adjunct in corneal transplantation. However, key gaps remain. No peer-reviewed clinical trial data are available, and the optimal human dosing regimen, preclinically between 0.4% and 2.0% applied three times daily, has not been established. Systemic exposure appears negligible, but local adverse effects (notably conjunctival hyperaemia and epithelial oedema) could complicate postoperative care. Before clinical adoption, randomized trials combining ROCKI with standard corticosteroids and calcineurin inhibitors, and incorporating long-term endpoints such as graft clarity, endothelial cell density, and patient-reported outcomes, are essential.

## 11. Emerging Therapies (ROCKI + Cell Therapy, Sustained Delivery)

Synergistic approaches combining ROCKI with novel therapeutic modalities have been reported in the literature. In preclinical studies, inclusion of ROCKI has improved the engraftment of transplanted CEC, suggesting synergy in cell-based therapies for endothelial dysfunction [21]. The most prominent example is Kinoshita et al., where injection of cultured human CEC supplemented with Y-27632 into the anterior chamber restored corneal transparency [6]. A side-by-side rabbit study found that both a tissue-engineered endothelial sheet and a cell-injection approach restored clarity and pump function; Y-27632 was included in both delivery media [155]. Moreover, screening of newer ROCKI (belumosudil, ripasudil, fasudil) on primary HCEC shows comparable or superior cytoprotection and proliferation versus Y-27632 [156]. Similar strategies using induced-pluripotent-stem-cell–derived or umbilical-cord–derived CECs in combination with ROCKI have demonstrated recovery of corneal clarity in rabbit and primate models of endothelial diseases like FECD and PBK, through enhanced cell adhesion and proliferation in the host cornea [157,158]. Beyond whole-cell therapy, ROCKI are incorporated into scaffold-based endothelial grafts and cell-sheet transplants—such as plastic-compressed collagen matrices and scaffold sheets—to improve cell survival and monolayer function in ex vivo and animal models [159].

Sustained-release formulations are also under investigation to overcome the short half-life of topical ROCKI. Experimental systems include thin mucoadhesive polymeric films for extended Y-27632 delivery to the corneal surface, drug-loaded nanoparticles—such as nanoceria carriers—for improved ocular retention [160]. Preliminary studies have shown that incorporating ROCKI into biodegradable poly lactic/glycolic acid microspheres release the drug over 7–10 days, reducing dosing frequency [161]. Similarly, approaches using nanocarriers such as hollow mesoporous nanoceria are under investigation to maintain therapeutic ROCKI levels intraocularly [162]. Additional depot concepts—drug-eluting hydrogels, contact-lens reservoirs, and lens-capsule implants—remain pre-clinical but share the same goal of extending dosing intervals [163,164].

Other innovative approaches include combining ROCKI with anti-VEGF or corticosteroid agents in a single formulation—as described in recent patent filings—to target corneal neovascularization or improve graft integration [165]. Gene therapy strategies, such as adeno-associated virus serotype 2 mediated delivery of dominant-negative RhoA, aim to provide long-term Rho-kinase suppression with a single treatment [166].

## 12. Limitations and Future Directions

Despite substantial preclinical and early clinical data supporting the potential of ROCKI in its application across a range of corneal pathologies, the current evidence base remains limited to small series or short-term trials. Most therapeutic reports are non-randomized and underpowered. Therefore, effect sizes, optimal dosing and head-to-head comparisons remain uncertain. Robust clinical trials—especially randomized, controlled studies—are needed in indications like FECD, PBK, and post-surgical oedema. Follow-up duration has generally been limited to three to twelve months, leaving uncertainty surrounding its long-term efficacy, durability of response, and safety—particularly its impact on corneal nerve health and epithelial integrity. Although ocular adverse events have been infrequently reported, larger cohorts are needed to establish true incidence rates, identify risk factors, and refine management strategies.

Another limitation is isoform specificity. Mechanistically, all approved drugs inhibit both ROCK 1 and ROCK 2, although in vitro work with corneal stromal cells suggests that ROCK 2 plays the dominant role in extracellular-matrix remodelling, implying that isoform-selective inhibitors could improve efficacy and reduce off-target effects. Optimal exposure schedules (continuous versus pulse dosing) and rational combinations, such as pairing ROCKI with anti-VEGF, anti-fibrotic or corticosteroid therapy, also await controlled evaluation.

Drug delivery remains the other major translational hurdle. Frequent topical dosing poses a practical barrier to patient adherence, underscoring the need for rigorous evaluation of sustained-release platforms, including nanocarriers, contact lens inserts, and intracorneal depots, to optimize therapeutic exposure and biocompatibility.

Addressing these gaps will require adequately powered, multicentre randomized controlled trials of sufficient duration, alongside parallel development of isoform-selective molecules and patient-friendly delivery systems.

## 13. Conclusions

ROCK is a key regulator of corneal cellular behaviour, influencing cytoskeletal dynamics, proliferation, apoptosis, inflammation, and fibrosis. Inhibition of this pathway has emerged as a promising therapeutic strategy across a wide spectrum of corneal diseases. Preclinical and early clinical studies demonstrate that ROCKI can enhance endothelial wound healing, improve cell survival, reduce fibrosis, and modulate immune responses.

In conditions such as FECD and PBK, ROCKI—particularly when combined with cell-based or surgical interventions—promote accelerated recovery of endothelial function. Following cataract surgery, evidence suggests a reduction in endothelial cell loss, while in corneal transplantation models, they suppress neovascularization and lower the risk of allograft rejection. These multifaceted effects stem from ROCKI acting on common cellular pathways underlying corneal pathology.

Despite advances in our understanding of the therapeutic potential of ROCKI, clinical translation remains in its early stages. Most human studies are limited in size and duration, leaving key questions about optimal dosing, long-term safety, and patient selection unanswered. Realizing the full therapeutic potential of ROCKI will be aided by robust, randomized controlled trials to define its role in corneal care.

Integration with regenerative therapies, such as cultured endothelial cell transplantation, and sustained-release delivery platforms may further expand the clinical utility of ROCKI, offering less invasive, more durable treatment options. ROCKI has the potential to reshape corneal medicine—transforming surgical conditions into medically manageable diseases and improving outcomes for patients worldwide.

## Figures and Tables

**Table 1:** Overactivation and key effects of RhoA/ROCK signalling pathway in corneal biology. IL: interleukin. TNF- α: tumour necrosis factor alpha. TGF-β: transforming growth factor beta. α-SMA: alpha smooth muscle actin. DSO: Descemet stripping only. CEC: corneal endothelial cell. FECD: Fuch’s endothelial corneal dystrophy. TLR4: toll like receptor 4. Treg: regulatory T-cells. Th17: T helper 17. Na^+^/K^+^-ATPase: sodium–potassium adenosine triphosphatase. VEGF: vascular endothelial growth factor. ZO-1: zonula occludens-1 (ZO-1).

Corneal Layer	Basal Role of ROCK	Pathological Over-Activation	Key Effects of ROCK Inhibition
Epithelium	Coordinates cortical F-actin and adherens/tight junctions during sheet migration and barrier renewal	NF-κB-driven cytokine surge (IL-1β, IL-6, TNF-α) and leukocyte influx after infection or chemical injury	Y-27632 relaxes actomyosin, boosts migration/adhesion and closes scratch defects faster than control
Stroma (keratocytes /fibroblasts)	Couples TGF-β signalling to α-SMA expression, myofibroblast contractility and extracellular matrix deposition	Fibrotic haze and high-tension scars after surgery or burns	Topical or in-matrix Y-27632 blocks keratocyte-to-myofibroblast switch, normalises collagen to an embryonic-like lattice
Endothelium	Maintains hexagonal architecture and pump barrier by stabilising F-actin, ZO-1 and Na^+^/K^+^-ATPase	Oxidative/inflammatory stress → contraction, junction loss, caspase-3 apoptosis → oedema	Y-27632 / ripasudil relax cytoskeleton, up-shift cyclin-D, enhance spreading & Rac1-driven migration; accelerate clearance after DSO or CEC injection; reactivate FECD endothelia
Immune & inflammatory axis	ROCK 1 amplifies TLR4-NF-κB signalling	Persistent stromal inflammation, delayed healing	Inhibitors dampen cytokines, favour Treg over Th17, reduce graft rejection
Angiogenesis	Up-stream of VEGF and Shh–Rac pathways in limbal ECs	Pathologic corneal neovascularisation	Fasudil or netarsudil eye-drops reduce corneal neovascularization area in alkali-burn mice

**Table 2:** Summary of selected ROCKI, including their selectivity, formulation, key pharmacologic features, and preferred or investigational ocular indications. NET: norepinephrine transporter.

ROCKI	Selectivity	Formulation/Administration	Key Pharmacologic Features	Preferred/Investigated Indications
Netarsudil	Non-selective (ROCK1 & ROCK2) + NET inhibitor	0.02% topical solution, once daily	Dual ROCK and NET inhibition; long corneal half-life	Glaucoma, ocular hypertension, corneal endothelial disorders, post-surgical recovery
Ripasudil	Non-selective (ROCK1 & ROCK2)	0.4% topical solution, twice daily	Rapid corneal uptake; shorter corneal effect (~6 h); rapid systemic clearance	Glaucoma, corneal endothelial regeneration, wound healing
Fasudil	Non-selective (ROCK1 & ROCK2)	Intravenous (approved in Japan for cerebral vasospasm); intravitreal in trials	ATP-competitive inhibitor; reversible binding; off-target at high dose	Cerebral vasospasm, retinal edema (in trials)
Y-27632	Non-selective (ROCK1 & ROCK2)	Experimental; topical in pre-clinical/animal studies	ATP-competitive inhibitor; nanomolar potency; off-target at high dose	Pre-clinical glaucoma and corneal research
SNJ-1656 (Y-39983)	Non-selective (ROCK1 & ROCK2)	Experimental; topical in pre-clinical/animal studies, Phase II for glaucoma	More potent derivative of Y-27632	Glaucoma (Phase II), corneal disease (experimental)

**Table 3:** Key studies of ROCKI in FECD. In all, topical ROCKI (mainly ripasudil) have been associated with high clearance rates and vision gains, supporting their role as a viable non-graft therapy for select FECD patients. EMT: epithelial–mesenchymal transition. CCT: central corneal thickness. BCVA: best corrected visual acuity. ECD: endothelial cell density. CDVA: corrected distance visual acuity. Na^+^/K^+^-ATPase: sodium–potassium adenosine triphosphatase. ZO-1: zonula occludens-1 (ZO-1). ↑: increase/upregulated. ↓: decrease/downregulated.

Study (Year, Ref.)	Design	Subjects/Model (*n*)	Intervention & Dosing	Key Findings	Follow-up
Schlotzer-Schrehardt et al. (2021) [7]	Experimental (ex vivo/in vitro)	FECD lamellae (*n* = 450), wound model (*n* = 30), intact (*n* = 20), FECD cell lines (*n* = 3)	Single 30 µM ripasudil dose	↑ Cell-cycle, adhesion/migration, barrier & pump genes/proteins; ↓ EMT markers in FECD & normal samples	24–72 h
Parekh et al. (2024) [23]	Experimental (ex vivo DSO model & in vitro)	Immortalized CECs from cadaveric normal and FECD donors	Ripasudil (0.3 µM, 1 µM, and 10 µM)	Enhanced migration & wound closure	24–72 h
Okumura et al. (2013) [18]	Preclinical + clinical case series	Monkeys (*n* = 7); human corneal oedema (central *n* = 4, diffuse *n* = 4)	Y-27632 drops 6×/day	Central oedema: CCT ↓ at 6 months; Diffuse oedema: no CCT change; ED density: ↑ to ~3000 cells/mm^2^ with restored ZO-1 & Na+/K+-ATPase	4 weeks for primates, 6 months for humans
Lindstrom et al. (2022) [96]	Phase 2 RCT, open-label, parallel-group	FECD patients (*n* = 40; CCT ≥ 600 µm, BCVA 20/40–20/400)	Netarsudil 1×/day vs. 2×/day for 8 weeks	At week 4: CCT ↓ 28.4 µm (x1/day dosing), ↓ 20.1 µm (x2/day dosing); 12.5% oedema resolution; 25% gained ≥ 10 letters; benefits persisted to week 8; no significant differences in dosing groups; well tolerated	8 weeks
Moloney et al. (2021) [8]	Pre-post clinical with historical control	FECD eyes with DSO (*n* = 23)	Ripasudil 0.4% 6×/day until clearance	96% (22/23) cleared at mean 4.1 weeks; VA +0.20 LogMAR, BCVA +0.156; 1 failure	12 months
Davies et al. (2021) [92]	Intra- & inter-patient pilot	FECD eyes with DSO + cataract surgery (*n* = 20 eyes, 10 patients)	Netarsudil immediately vs. delayed post-DSO	Immediate use: clearance 4.6 ± 1.7 weeks vs. 8 ± 1.9 weeks (*p* < 0.01); “rescue” clearance 1–2 weeks after; higher ECC with immediate use (*p* = 0.05)	Until clearance
Macsai & Shiloach (2019) [93]	Prospective (non-randomized controlled)	FECD patients DSO ± cataract (ripasudil *n* = 9 vs. control *n* = 9)	Ripasudil 0.4% 4×/day for 2 months vs. none	Clearance 4.6 vs. 6.5 weeks (*p* < 0.01); ripasudil group maintained peripheral ECD (no change), controls lost 10% ECD by 12 months (*p* < 0.05)	12 months
Price et al. (2021) [97]	Prospective, randomized, double-masked pilot trial	FECD patients (*n* = 29)	Netarsudil 0.02% vs. placebo for 3 months	CCT reduction: –20 µm at 1 month; –26 µm at 3 months; scotopic CDVA + 1.6 lines; no change in disability scores; one withdrew (glare)	3 months

## Data Availability

No new data were created.

## Abbreviations

The following abbreviations are used in this manuscript:

ROCK	Rho-associated protein kinase
ROCKI	Rho-associated protein kinase inhibitor
CEC	Corneal endothelial cell
FECD	Fuchs endothelial corneal dystrophy
PBK	Pseudophakic bullous keratopathy
RBD	Rho-binding domain
MLC	Myosin light chain
MYPT1	Myosin phosphatase target subunit
α-SMA	Alpha smooth muscle actin
DSO	Descemet stripping only
NF-κB	Nuclear factor kappa B
IL	Interleukin
TNF-α	Tumour necrosis factor alpha
TGF-β	Transforming growth factor beta
TLR4	Toll like receptor 4
Treg	Regulatory T-cells
Th17	T helper 17
VEGF	Vascular endothelial growth factor
IOP	Intraocular pressure
NET	Norepinephrine transporter
DSAEK	Descemet stripping automated endothelial keratoplasty
EK	Endothelial keratoplasty
DMEK	Descemet membrane endothelial keratoplasty
EMT	Epithelial–mesenchymal transition
CCT	Central corneal thickness
BCVA	Best corrected visual acuity
ECD	Endothelial cell density
CDVA	Corrected distance visual acuity
ICE	Iridocorneal endothelial syndrome
Na^+^/K^+^-ATPase	Sodium–potassium adenosine triphosphatase
ZO-1	Zonula occludens-1
